# Current Progress and Future Perspectives of Immune Checkpoint in Cancer and Infectious Diseases

**DOI:** 10.3389/fgene.2021.785153

**Published:** 2021-11-30

**Authors:** Xin Cai, Huajie Zhan, Yuguang Ye, Jinjin Yang, Minghui Zhang, Jing Li, Yuan Zhuang

**Affiliations:** ^1^ Heilongjiang Administration of Traditional Chinese Medicine, Harbin, China; ^2^ Department of Pathology, Harbin Medical University, Harbin, China; ^3^ Department of Gynecology, Harbin Medical University Cancer Hospital, Harbin, China; ^4^ Department of Oncology, Chifeng City Hospital, Chifeng, China; ^5^ Department of Pathology and Electron Microscopy Center, Harbin Medical University, Harbin, China

**Keywords:** immune checkpoint, immunotherapy, cancer, microbiome, PD-1/PD-L1

## Abstract

The inhibitory regulators, known as immune checkpoints, prevent overreaction of the immune system, avoid normal tissue damage, and maintain immune homeostasis during the antimicrobial or antiviral immune response. Unfortunately, cancer cells can mimic the ligands of immune checkpoints to evade immune surveillance. Application of immune checkpoint blockade can help dampen the ligands expressed on cancer cells, reverse the exhaustion status of effector T cells, and reinvigorate the antitumor function. Here, we briefly introduce the structure, expression, signaling pathway, and targeted drugs of several inhibitory immune checkpoints (PD-1/PD-L1, CTLA-4, TIM-3, LAG-3, VISTA, and IDO1). And we summarize the application of immune checkpoint inhibitors in tumors, such as single agent and combination therapy and adverse reactions. At the same time, we further discussed the correlation between immune checkpoints and microorganisms and the role of immune checkpoints in microbial-infection diseases. This review focused on the current knowledge about the role of the immune checkpoints will help in applying immune checkpoints for clinical therapy of cancer and other diseases.

## Introduction

Activation of T cells plays an important role in the process of immunity (Lenschow and Bluestone, 1993). During normal immune response, the process that T cells accept antigen peptides presented by major histocompatibility complex (MHC) on antigen-presenting cells (APCs) *via* T-cell receptor (TCR) in order to exert its function is called the first signal for T-cell activation. The second signal for T-cell activation is a costimulatory signal which comes from a combination between CD28 on T cells and CD80(B7-1)/CD86(B7-2) on APCs (Lenschow et al., 1996; Nandi et al., 2020). This activation process also requires cytokines such as IL-2 to help. The rightly activated T cells or in tandem with B cells will eliminate threats, while uncontrolled activation of T cells would bring serious consequences such as autoimmune diseases (Takeuchi et al., 2020). Therefore, scientists devoted their lives to shed light on how the immune system regulates itself.

**FIGURE 1:**
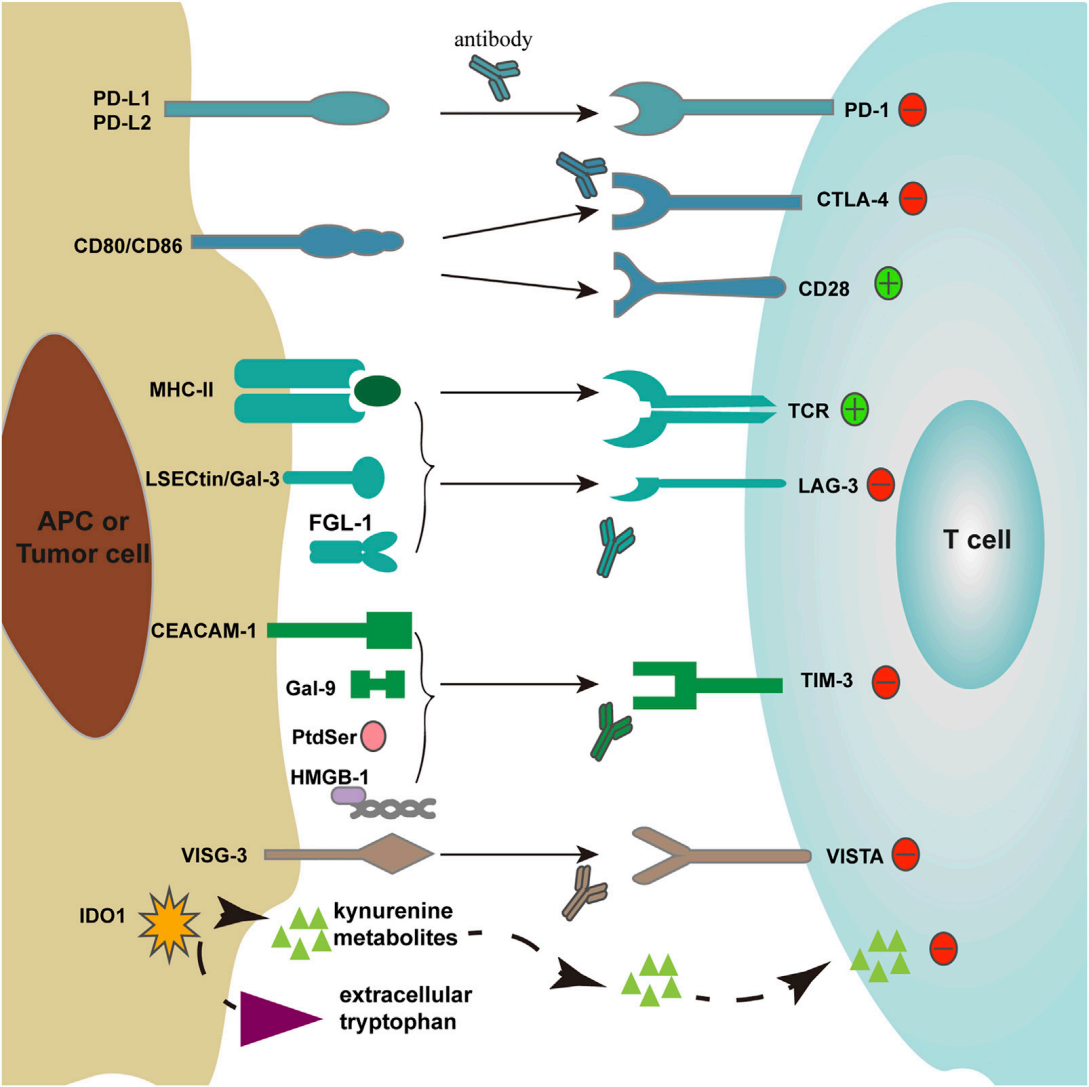
Immune checkpoint receptors and their ligands. Two signals participate in T-cell activation: 1) T cells recognize antigen presented by MHC-II molecules on APCs through TCR; 2) T cells accept costimulatory signals CD80/CD86 through CD28.

In the last two decades, the understanding of regulatory pathways in immune responses to cancer immunotherapies remains unclear. The enormous progress was made in 1996; Leach and his colleagues (Linsley et al., 1991; Leach et al., 1996) have been validated that blockade of cytotoxic T-lymphocyte-associated antigen 4 (CTLA-4) could downregulate T-cell responses and enhance antitumor responses in immunocompetent mouse models. In 2000, Gordon J. Freeman identified that CTLA-4 structurally similar protein-programmed death 1 (PD-1) could bind to its ligand PD-L1 and lead to the inhibition of lymphocyte proliferation (Freeman et al., 2000). The binding of B- and T-cell lymphocyte attenuator (BTLA) to its ligand HVEM may lead to decreased T-cell proliferation and cytokine production (Murphy et al., 2006). The binding of T-cell immunoglobulin and mucin domain-containing 3 (TIM-3) to its ligand galectin-9 could result in T helper 1 (Th1) cell death (Zhu et al., 2005). V-domain Ig suppressor of T-cell activation (VISTA) is a potent T-cell suppressor and inhibits T-cell immune response in animal models (Wang et al., 2011). During these processes, the set of costimulatory or coinhibitory molecules, which regulate the activation, effector functions, and interactions among APCs and T lymphocytes, provides a critical checkpoint in the regulation of T-cell immunity and maintenance of immune homeostasis. As their function in the balance of the immune system, these costimulatory or coinhibitory proteins are defined as immune checkpoint proteins (Figure 1, Table 1). A direct consequence of these findings was to reveal the regulatory pathways involved in immune responses in cancer and infectious diseases.

**TABLE 1:** The expression and mechanism of the immune checkpoints.

Immune checkpoints	Expression	Ligand	Mechanisms	PMID
PD-1	Activated T cells, Tregs, B cells, NK cells, DCs, macrophages, and monocytes	PD-L1 and PD-L2	ITSM recruits SHP-2, which acts as a bridge between two PD-1 molecules and induces inhibitory function of PD-1	30851633, 32184441, and 28443090
CTLA-4	Activated T cells and Tregs	CD80 and CD 86	Conserved YVKM motif in the cytoplasmic tail of CTLA-4 mediates recruitment of SH2-domain-containing proteins to regulate immune response	10411922, 18845758, and 29794465
LAG-3	Activated T cells, Tregs, NK cells, DCs, and B cells	MHC-II, LESCtin, Galectin-3, FGL-1, and α-synuclein	The KIEELE motif is considered to be essential for LAG-3 mediated inhibition	33488626 and 34067904
TIM-3	Activated T cells, TH17 cells, Tregs, DCs, NK cells, and monocytes	Galectin-9, CEACAM-1, HMGB-1, and PtdSer	TIM-3 exerts its function through several tyrosine residues	29069302 and 31676858
VISTA	Myeloid cell, T cells, and Tregs	VSIG-3 and PSGL-1	VISTA has the potential function of both a receptor and a ligand. The precise mechanism of VISTA needs to be explored	29375120 and 31690319
IDO1	A heme-containing enzyme participates in tryptophan metabolism	L-Tryptophan	Accumulation of kynurenine metabolites leads to suppression of T cells and induction of Tregs	20720200 and 33883013

Immune checkpoint proteins have been playing a significant role in inflammatory reactions and cancer immunotherapy. A number of immune checkpoint proteins were shown to be dysregulated in cancers and infectious diseases, including PD-1/PD-L1, CTLA-4, lymphocyte activation 3 (LAG-3), TIM-3, VISTA, and Indoleamine-2,3 dioxygenase 1 (IDO1). These immune checkpoints and other regulatory cells, such as regulatory T cells (Tregs), myeloid-derived suppressors cells (MDSCs), M2 macrophages, and cytokines, are often enhanced during infections and cancers (Pauken and Wherry, 2015). Pathogens can develop immune checkpoints to limit host-protective antigen-specific immune response (Dyck and Mills, 2017). The cancer cells can disrupt the immune response and cleverly escape from immunity by dysregulating immune checkpoint signaling. Many similarities exist between cancer and infectious disease (Hotchkiss and Moldawer, 2014). They can utilize similar receptors to detect damage-associated molecular patterns (DAMP) and pathogen-associated molecular patterns (PAMPs), respectively (Vance et al., 2017). In the meantime, persistent stimulation of the immune system and induction of T-cell-mediated inflammation can be aroused. In pathogen-infected diseases, with elevated expression of the immune checkpoint molecules on T cells as it is in cancer, the immune checkpoint blockade therapy may bring favorable consequences (Wykes and Lewin, 2018). So, agonists of costimulatory signals or antagonists of inhibitory signals function as good ways for cancer therapy and also could help to reverse the state of immune suppression in chronic infection. Some antibodies that targeted immune checkpoint molecules to reverse the suppression of the immune system have been applied in the clinical treatment of cancer (Remon and Besse, 2017; Chen et al., 2019). However, the unexpected events of an immune checkpoint inhibitor (ICI) have emerged as frequent complications at the same time.

Here, we review the mechanisms, functions, and adverse events of common immune checkpoints in cancer and infectious diseases. We also discuss the impact of the bacterial microbiome on the relationship between cancer therapy and the immune system.

## Biology of Immune Checkpoint Proteins

### PD-1/PD-L1

PD-1 is a 288 amino acid protein that is encoded by the *PDCD1* gene and belongs to the immunoglobulin superfamily (Tavares et al., 2018). PD-1 can be expressed on T cells, B cells, natural killer cells (NKs), dendritic cells (DCs), macrophages, and monocytes (Ahmadzadeh et al., 2009). T cells inducibly express PD-1 after activation (Han et al., 2020), while different from other members of the CD28 superfamily, which has Src homology (SH2) binding motifs and/or SH3 binding motifs in their cytoplasmic tail, the cytoplasmic tail of PD-1 possesses a sequence that can form an immunoreceptor tyrosine-based inhibition motif (ITIM) and an immunoreceptor tyrosine-based switch motif (ITSM) that can recruit Src homology 2 domain-containing protein tyrosine phosphatases (SHP-2), resulting in the inhibitory function (Neel et al., 2003; Patsoukis et al., 2020).

The two ligands of PD-1, PD-L1 (also known as B7-H1) and PD-L2 (B7-H2), differ in expression patterns (Panjwani et al., 2018). PD-L1 is expressed on many cells, including B cells, T cells, macrophages, tumor cells, and other tissue cells such as vascular endothelial cells (Ritprajak and Azuma, 2015; Dermani et al., 2019). Ligation of PD-1 and PD-L1 can lead to T-cell dysfunction and anergy, helping PD-L1 expressing tumor cells escape from cytotoxic T-cell-mediated cell death (Ritprajak and Azuma, 2015; Dermani et al., 2019).

PD-1/PD-L1 blockade not only facilitates T-cell function but also restores NKs antitumor response (Hsu et al., 2018). PD-L1 expression on cancer cells resulted in the generation of more aggressive tumors *in vivo*. Depleting NKs before PD-L1 expressed or not tumor cell implantation resulted in similar growth of tumors and mortality. However, no such effect occurring with depletion of CD4+ and CD8+ T cells indicates that NKs take a vital position in immune checkpoint blockade (Hsu et al., 2018).

It is reported that several signaling pathways would participate in the PD-1/PD-L1 axis. For example, PD-1^−^PD-L1^+^ regulatory B cells must exert their immunosuppressive function through activation of the PI3K/AKT/NF-κB signaling pathway in breast cancer (Liu et al., 2021). PTEN is a critical inhibitor of the PI3K/AKT signaling pathway. In microsatellite instability-high (MSI-H) or mismatch repair deficient (dMMR) gastrointestinal tumors, mutation of PTEN, especially in the phosphatase domain, could be negative predictors of PD-1 blockade treatment (Chida et al., 2021). Blockade of MAPK pathway through MEK1 and two inhibitors prevented the expression of PD-L1 in lung adenocarcinoma cells (Stutvoet et al., 2019), whereas inhibition of ERK could improve the anti-PD-L1 checkpoint blockade effect in preclinical pancreatic ductal adenocarcinoma (Henry et al., 2021). What we have listed above indicates that MAPK pathway activity could also severely influence the PD-L1 axis despite the PI3K pathway. Similarly, using inhibitors of the JAK/STAT pathway, which was reported to suppress PD-L1 upregulation, showed that it can also take part in regulating the PD-L1 axis (Doi et al., 2017).

### CTLA-4

CTLA-4 is a 223 amino acid protein, which belongs to the immunoglobulin superfamily and consists of an IgV domain, a transmembrane region, and a cytoplasmic tail containing a conserved YVKM motif (Rowshanravan et al., 2018). Stored in endocytic vesicles, CTLA-4 is transported to the cell membrane to be colocalized with TCR on the cell surface. Dependent on dynamin and clathrin adaptor protein complex (AP2), which targets the YVKM motif, internalization of CTLA-4 from cell surface for degradation and recycling is rapid (usually within minutes) (Shiratori et al., 1997). Then CTLA-4 can be transported to cell membrane again or compartment of lysosome for degradation. Such regulation of AP2 can be disrupted by the phosphorylation of the YVKM motif after T-cell activation (Qureshi et al., 2012). Lipopolysaccharide responsive and beige-like protein (LRBA) may inhibit degradation of CTLA-4 by disrupting transportation of CTLA-4 to the lysosome via binding to YVKM sequence and promote recycling of CTLA-4. Patients with LRBA deficiency raised autoimmunity syndrome designating that accurate CTLA-4 trafficking is important for autoimmune diseases (Lo et al., 2015; Rowshanravan et al., 2018).

The phenomenon that CTLA-4, often expressed on antigen-specific T cells, has a higher affinity (10–100-fold) for CD80 dimer and CD86 monomer than CD28 is considered to be a conventional concept about how CTLA-4 downregulates the immune response (Linsley et al., 1994; van der Merwe et al., 1997). Different from antigen-specific T cells that upregulated CTLA-4 after activation, Tregs constitutively express a high range of CTLA-4 ensuring immune homeostasis and immunosuppressive capacity. Intriguingly, there have been studies proved that CD80 and CD86 on APC can be captured and deleted by CTLA-4 expressed on CD4+CD25+Foxp3+ Tregs (Qureshi et al., 2011; Tekguc et al., 2021), while patients or carriers with CTLA-4 mutation showed diminished Tregs inhibitory function and impaired trans-endocytosis of CD80 (Schubert et al., 2014). These discoveries provide a proper explanation for the rapid endocytic behavior of CTLA-4 that CTLA-4 may exhibit its inhibitory function by trans-endocytosis. Also, there have been studies about other mechanisms undergoing CTLA-4 inhibition. Kong et al. found that protein kinase C-η (PKC-η) was recruited to and physically associated with the CTLA-4 expressed on Tregs in the immunological synapse. PKC-η-deficient Tregs lacked their suppressive function, leading to lymphoproliferation and autoimmune syndromes (Kong et al., 2014). In addition, competitively binding with CD28, CTLA-4 limited the positive costimulation of CD28 by blocking the downstream PI3K/AKT and NF-κB signaling pathway (Pages et al., 1994; Olsson et al., 1999). The anti-CTLA-4 antibody (ipilimumab) eliminated Tregs in an Fc-dependent manner to achieve clinical relief, which may be due to relieved NKs cytotoxicity suppressed by Tregs (Romano et al., 2015; Khan et al., 2020). For anti-CTLA-4 antibodies therapy, CD8+ T cells were required for the therapeutic effect. Fas-FasL and perforin interactions also were important for CTLA-4 blockade (van Elsas et al., 2001).

### LAG-3

Firstly identified in 1990 by Triebel and colleagues, lymphocyte activation 3 (LAG-3, CD223), an immune inhibitory receptor, is a 503 amino acid protein encoded by lymphocyte activation gene that is located on chromosome 12, containing eight exons (Triebel et al., 1990; Sierro et al., 2011). Belonging to the Ig superfamily, LAG-3 contains four extracellular Ig-like domains D1, D2, D3, and D4, which share approximately 20% amino acid homology with that of CD4. Comprising unlike intracellular region with CD4, LAG-3 is closely related but exhibits divergent functions with CD4 (Maruhashi et al., 2020). The cytoplasmic tail of LAG-3 has three conserved motifs. The first motif, which has not been considered functional, contains a hypothesized serine phosphorylation site containing two serine residues in humans. It is reported that the second motif, which has conserved six amino acid sequences (KIEELE), plays an important role in dampening T-cell proliferation, cytokine production, and cytolytic function. The third motif is a glutamic acid and proline dipeptide repeat which can colocalize LAG-3 with CD3, CD4, and CD8 molecules (Goldberg and Drake, 2011; Ruffo et al., 2019).

LAG-3 can be detected from CD4+ and CD8+ T cells, Tregs, NKs, and plasmacytoid DCs and do not express on naive T cells similar to PD-1 and CTLA-4 (Goldberg and Drake, 2011). Activation of LAG-3 can elevate intratumoral Tregs activity, and blocking of it will upregulate T-cell function and reinvigorate CD8+ tumor-infiltrating lymphocytes (TILs) to eliminate tumor cells (Lecocq et al., 2020). CD4+CD25+ Tregs from LAG-3 (−/−) mice exhibited reduced regulatory activity. Treated with anti-LAG-3 antibody, suppression induced by Tregs was inhibited *in vitro* and *in vivo*. It is obvious that LAG-3 marks Tregs populations and intermediates their regulatory function (Huang et al., 2004). As a transmembrane protein receptor which is similar to CD4 with greater affinity for MHC-II molecules on APCs (Triebel et al., 1990), there are also other proposed ligands for LAG-3 like galectin-3, fibrinogen-like protein 1 (FGL-1), α-synuclein, and LSECtin (Xu et al., 2014; Kouo et al., 2015; Mao et al., 2016). Recent research showed that FGL-1 worked as an important ligand of LAG-3 in its inhibitory effect on T cells. The expression of LAG-3 can be elevated on exhausted T cells in cancer. FGL-1 is upregulated in several human cancers, and genetic ablation or blockade of the FGL-1/LAG-3 interaction by monoclonal antibodies (mAbs) would enhance T-cell responses and antitumor immunity. Wang et al. expected a poor prognosis in non-small-cell lung cancer (NSCLC) patients with high plasma FGL-1 treated with anti-PD therapy (Wang et al., 2019a). The precise function of ligands of LAG-3 still needs to be clarified.

### TIM-3

TIM-3 is a transmembrane protein encoded by *HAVCR2* and identified on IFN-γ-producing CD4+ Th1 cells and CD8+ type 1 cytotoxic T cells firstly. Then it is also discovered on monocytes, Tregs, DCs, and NKs (Wolf et al., 2020). The fact that administration of antibody to TIM-3 could enhance Th1-dependent autoimmune disease strongly implying that TIM-3 works as an inhibitory molecule on T-cell function (Monney et al., 2002). Indeed, TIM-3 is found to be coregulated and coexpressed with other immune checkpoint receptors, such as PD-1 and LAG-3 (Chihara et al., 2018). High expression of TIM-3 on effector T cells also indicates severe T-cell exhaustion or dysfunction (Avery et al., 2018).

Without known inhibitory signaling motifs in its cytoplasmic tail, TIM-3 contains five conserved tyrosines to play its role. TIM-3 can be found in lipid rafts and is recruited to the immunological synapse upon T-cell activation (Clayton et al., 2014). TIM-3 interacts with HLA-B associated transcript (BAT3) in ligand unbound form and maintains T-cell activation by recruiting an active form of tyrosine kinase LCK, while in ligand-bound form, tyrosine phosphorylation in its cytoplasmic tail will release BAT from TIM-3 and recruit tyrosine kinase FYN resulting in immune synapse disruption, phosphatase recruitment, and cell apoptosis (van de Weyer et al., 2006; Rangachari et al., 2012).

It has been demonstrated that IL-27/NFIL3 axis promotes permissive chromatin remodeling of the TIM-3 locus, induces TIM-3 expression, and is crucial for the induction of TIM-3 *in vivo*. IL-27-conditioned Th1 cells exhibit inhibitory function through NFIL3 in intestinal inflammation (Zhu et al., 2015). In human acute myeloid leukemia (AML), activation of TIM-3 works through NF-κB and β-catenin signaling pathways to promote self-renewal of leukemic stem cells (Kikushige et al., 2015). In hepatocellular carcinoma (HCC), TIM-3 was significantly upregulated in NKs and suppressed their cytokine production and cytotoxic activity through inhibiting PI3k/Akt/mTORC1 signaling pathway (Tan et al., 2020).

Different ligands of TIM-3 show various effects. The well-studied ligands of TIM-3 are galectin-9 (Gal-9), carcinoembryonic antigen-related cell adhesion molecule 1 (CEACAM-1), high mobility group box-1 protein (HMGB1), and phosphatidylserine (PtdSer). In T cells, ligation between Gal-9 and carbohydrate motifs on the IgV domain of TIM-3 functions in an immunosuppressive way which will induce T-cell apoptosis (Du et al., 2017; Dixon et al., 2018). CEACAM-1 coexpressed with TIM-3 is considered to be required for the regulatory function of TIM-3 (Huang et al., 2015). HMGB1 can bind to DNA released from dying cells and facilitate the uptake of DNA by Toll-like receptors. The interaction between HMGB1 and TIM-3 interferes with the innate immune response induced by nucleic acid (Nogueira-Machado et al., 2011; Chiba et al., 2012; Urban-Wojciuk et al., 2019). PtdSer-TIM-3 interaction shows clues for participating in apoptotic clearance cells, and more consequences between their interaction are waiting to be found (Nakayama et al., 2009).

Gal-9 binding with TIM-3 can cause an influx of calcium and mediate aggregation and apoptosis of effector Th1 cells *in vitro*. Administration of Gal-9 can result in selective loss of IFN-γ-producing cells and suppression of Th1 autoimmunity (Zhu et al., 2005). PtdSer engagement will induce TIM-3 phosphorylation leading to dysfunction of NKs in HCC (Tan et al., 2020). In head and neck squamous cell cancer (HNSCC), blockade of TIM-3 by mAbs induced the reduction of Tregs and increased IFN-γ production of CD8+ T cells, while the population of CD206+ M2 macrophages was not significantly reduced (Liu et al., 2018). Intriguingly, TIM-3 can also play an immunostimulatory role in NKs, DCs, and macrophages (Gleason et al., 2012; Zhang et al., 2012; Yang et al., 2013; Clayton et al., 2014).

### VISTA

V-domain Ig suppressor of T cell activation (VISTA), also termed as PD-1H, B7-H5, V-set immunoregulatory receptor (VSIR), stress-induced secreted protein 1 (SISPQ), and differentiation of embryonic stem cells 1 (Dies1), is a conventional transmembrane protein whose IgV domain homology with PD-L1 and encoded by the gene located on chromosome 10 (Huang et al., 2020). Although containing a similar molecular sequence with the B7 superfamily, VISTA does not possess ITIM/ITAM (immunoreceptor tyrosine-based activation motif). VISTA is expressed on myeloid cells (e.g., monocytes, conventional DCs, macrophages, and circulating granulocytes), T cells, Tregs, and TILs (Hosseinkhani et al., 2021). There are increasing pieces of evidence showing VISTA as a regulatory immune checkpoint. In mice lacking VISTA, they would develop spontaneous T-cell activation, cutaneous lupus erythematosus, and production of inflammatory cytokines and chemokines (Wang et al., 2014; Liu et al., 2015; Han et al., 2019). With the presence of VISTA on erythroid cells, the transformation from naive CD4+ T cells to Tregs would be accelerated through the production of TGF-β (Shahbaz et al., 2018).

Though the binding pattern of VISTA is not clear, several studies showed that VISTA could act as both ligand on APCs and receptor on T cells (Flies et al., 2014; Lines et al., 2014). Researches have reported V-Set and Immunoglobulin domain containing 3 (VSIG-3) as the ligand for VISTA in impeding cytokine and chemokine production (Wang et al., 2019b). In consideration of elevated expression of VISTA or VSIG-3 in many cancers, such as colorectal cancer (CRC), HCC, and intestinal-type gastric cancers, the blockade of the VISTA/VSIG-3 pathway can work as a new target for immune checkpoint therapy. Besides, Alan et al. presented that VISTA can bind to P-selectin glycoprotein ligand-1 (PSGL-1) in a pH-dependent model (Johnston et al., 2019). Meanwhile, a study of VISTA in malignant pleural mesothelioma shows that VISTA expression was associated with better overall survival (OS), suggesting VISTA’s prognostic value (Muller et al., 2020).

### IDO1

Indoleamine-2,3 dioxygenase 1 (IDO1) is one of the three enzymes which catalyze the first rate-limiting step in the oxidative metabolism of tryptophan, an essential amino acid for T-cell proliferation and differentiation. It is mainly distributed in DCs, macrophages, and monocytes (Munn and Mellor, 2013).

Tumor cells can recruit IDO-expressed DCs into the tumor microenvironment (TME). Due to the aggregation of IDO, lack of tryptophan will lead to stagnation of T-cell proliferation and differentiation in many ways. First, decreased tryptophan means elevated uncharged Trp-tRNA, which leads to activation of a stress response kinase, general control nonderepressible 2 (GCN2) (Munn et al., 2005). Then eukaryotic initiation factor-2 (eIF-2) is phosphorylated by GCN2, and translation of protein required for generation and proliferation of effector T cells will be limited. Second, degradation of tryptophan results in suppression of mammalian target of rapamycin complex 1 (mTORC1) and PKC-θ associated with induction of autophagy. Apoptosis of effector T cells will be reinforced (Metz et al., 2012). Third, IDO1 can induce Tregs through increased activity of aryl hydrocarbon receptor (AHR) binding with kynurenine, a metabolite of tryptophan (Mezrich et al., 2010). Thus, the unbalanced metabolism of tryptophan can promote tumor development and evade immune detection indicating that the application of IDO1 inhibitor is also a promising means to enhance antitumor immunity in theory. In status quo, clinical application of IDO1 inhibitor displayed a controversial outcome with rare effect on monotherapy and combination therapy. Although the agents might not be suitable for such types of cancer involved in research, they may be helpful in other diseases.

## Single Agent and Combined Therapy in Cancer

Balckburn et al. have demonstrated that T-cell function decreases with increased expression of immune checkpoints, so targeting these immune checkpoint proteins to modulate immune responses holds great promise for cancer immunotherapy (Blackburn et al., 2009). The purpose of immune checkpoint blockade is mainly to suppress CD8+ T cells and improve tumor-specific immune response. The mAbs by targeting checkpoints CTLA-4 and PD-1/PD-L1 have achieved the US Food and Drug Administration (FDA) approval for the treatment of different cancers (Peggs et al., 2006; Hodi et al., 2010).

Ipilimumab was the first FDA-approved recombinant humanized anti-CTLA-4 immunoglobulin G1 monoclonal antibody in 2011 for the treatment of advanced melanoma in patients who cannot be surgically cured or have metastasis (Vaddepally et al., 2020). It can also work well with intermediate or poor-risk advanced renal cell carcinoma (RCC), MSI-H/dMMR CRC, metastatic NSCLC, unresectable malignant pleural mesothelioma, and HCC, which have been previously treated with sorafenib, in combination with nivolumab (Pinto et al., 2019; McKay et al., 2020; Baas et al., 2021; Casak et al., 2021; Saung et al., 2021). In 2014, nivolumab and pembrolizumab (PD-1 blockade) were approved by the FDA as a humanized IgG antibody for the treatment of unresectable or metastatic melanoma (Prasad and Kaestner, 2017; Finkelmeier et al., 2018). In 2016, the PD-L1 blockade, atezolizumab, a humanized IgG antibody, officially worked as a second-line treatment for locally advanced or metastatic urothelial carcinoma (Patel et al., 2017). With the maturity of theory and technology, the usage range of PD-1/PD-L1 blockade has gradually expanded, including metastatic nonsquamous NSCLC, advanced RCC, unresectable or metastatic, recurrent HNSCC, MSI-H/dMMR CRC, relapsed or refractory classical Hodgkin lymphoma (cHL), locally advanced or metastatic urothelial carcinoma, cervical cancer, gastric cancer, and esophageal cancer (Ansell et al., 2015; Beckermann et al., 2017; Chae et al., 2018; Lin et al., 2018; Saito et al., 2018; Oliveira et al., 2019; Wang and Li, 2019; Nassar et al., 2020; Wu et al., 2020). Pembrolizumab and nivolumab targeting PD-1 showed promising results in melanoma and NSCLC with an objective response rate (ORR) of 40–45% (Darvin et al., 2018). LAG-3 is coexpressed with many inhibitory immune checkpoints, especially PD-1, and this signifies a more exhausting state than expressing PD-1 alone. Utilization of coblockade for PD-1 and LAG-3 shows better curative effects. Relatlimab (in combination with nivolumab) is the first LAG-3 blocking antibody to demonstrate a benefit for patients in a Phase 3 study (Lipson et al., 2021). IMP321, a recombinant soluble LAG-3 Ig fusion protein of which multiple phases I and phase II trials have been completed, may enhance T-cell response, expand the percentage of long-lived effector-memory CD8+ T cells, and rarely induce immune-related adverse events (irAEs) (Brignone et al., 2009; Wang-Gillam et al., 2013). TIM-3, as an immunoinhibitory molecule, indicates the most terminal state of T cells, whose antibodies are being studied and evaluated for clinical trials, including Sym023 (NCT03489343), TSR-022 (NCT03680508), LY3321367 (NCT03099109), and MBG453 (NCT02608268). Many studies focus on the combination between anti-TIM-3 antibody and anti-PD-1 antibody in patients with advanced relapsed or a refractory solid tumor. There are also some ongoing clinical trials that evaluate the safety and feasibility of different ICIs in various tumors. Therapeutically targeting BTLA, VISTA, TIM-3, and TIGIT remain in preclinical stages to treat advanced solid malignancies (Derre et al., 2010) (NCT02671955, NCT02817633, NCT02608268, and NCT03119428).

The combination of immune checkpoints may improve clinical response rates. CTLA-4 and PD-1 blockade combination could increase effector T-cell infiltration into B16 melanoma in mice (Curran et al., 2010). Nivolumab plus ipilimumab in patients with metastatic melanoma yielded a response rate from 40% with treatment alone to 72% among patients who were PD-L1-positive (Larkin et al., 2015). In an open-label, randomized, phase 3 study (CheckMate 743), the results showed that nivolumab plus ipilimumab prolonged the median of the OS by nearly one-third versus chemotherapy (18.1 versus 14.1 months) and 2-years OS rates by nearly a half (41 versus 27%) (Baas et al., 2021). Early data using relatlimab plus nivolumab showed promising antitumor activity with an 11.5% ORR (NCT01968109). Now more and more researches focus on combination medication on relatlimab in HCC (NCT04658147), melanoma (NCT03743766), refractory MSI-H solid tumor (NCT03607890), HNSCC (NCT04326257), and so on. Although the clinical effectiveness of these ICIs gained great success in cancer immunotherapy, a subset of patients still does not respond to these inhibitors.

There are also some studies that showed that immune checkpoint blockade combined with radiotherapy, chemotherapy, and targeted drugs could improve the antitumor efficacy (Twyman-Saint Victor et al., 2015; Ebert et al., 2016; Shi et al., 2016). In the murine HCC model, combination with anti-TIM-3 and radiotherapy significantly shrink the tumor growth and elongate the OS compared with monotherapy (Kim et al., 2021). In an open-label, randomized, phase III trial (CheckMate 649), nivolumab plus chemotherapy reveals promising prospects than chemotherapy alone with superior OS and progression-free survival (PFS) benefit (Janjigian et al., 2021). Guidelines recommended using atezolizumab plus nab-paclitaxel for first-line treatment of unresectable, locally advanced, or metastatic triple-negative breast cancer (TNBC) with PD-L1 expressed on tumor-infiltrating immune cells. A survival analysis found that the OS, safety outcomes, and occurrence of immune-mediated adverse events of atezolizumab plus nab-paclitaxel were all ameliorated than placebo plus nab-paclitaxel (Emens et al., 2021). A TLR9 binding CpG-ODN adjuvant with a systemic anti-CTLA-4 antibody could increase the survival of mice bearing poorly immunogenic B16 melanoma (Davila et al., 2003).

## Immune-Related Adverse Events Induced by ICIs

As we know, immune checkpoint blockade has demonstrated a significant promise in the clinic across a range of cancer indications (Chen and Mellman, 2017). However, the immune checkpoint blockade can reinforce host immunity at an expanse of uncontrolled effects that results in a unique spectrum of toxicities defined as immune-related adverse effects (irAEs) (Xu et al., 2018). The degree of irAEs is divided into five grades, comprising mild, moderate, severe, life-threatening, and death, elucidated on Common Terminology Criteria for adverse events from US National Cancer Institute (Cancer Therapy Evaluation Program, 2017). Some key oncology societies recently published comprehensive guidelines for irAEs, including the American Society of Clinical Oncology (ASCO), the European Society for Medical Oncology (ESMO), the Society for Immunotherapy of Cancer Toxicity Management Working Group, and the National Comprehensive Cancer Network (Connolly et al., 2019; Ramos-Casals et al., 2020). The referred organs/system of irAEs include, but are not limited to, cardiac, dermatological, endocrine, gastrointestinal, neurological, muscular, pulmonary, ocular, renal, skeletal, and systemic toxicities.

Paolo et al. declared that irAEs occurring in patients treated with ipilimumab were dose-dependent (Ascierto et al., 2017). Generically, the earliest and the most frequent symptom that showed up during ICI therapy (both anti-CTLA-4 and anti-PD-1) was dermatological changes (Sandigursky and Mor, 2018). A meta-analysis of irAEs in phase III randomized controlled trials of lung cancer proposed that the most frequent irAEs were diarrhea, skin rash, and hypothyroidism (Berti et al., 2021). Another network meta-analysis specifically presented that the main irAEs of ipilimumab were related to the gastrointestinal system (diarrhea, 29%) and skin (rash, 31%), while nivolumab and pembrolizumab were referred to as less frequency in irAEs with maculopapular rash (13%), erythema (4%), hepatitis (3%), arthralgia (12%), hypothyroidism (8%), and hyperglycemia (6%), respectively (Almutairi et al., 2020). A retrospective analysis about North American Intergroup trial E1609 with 1,673 patients proclaimed that grade 1-2 irAEs were associated with longer relapse-free survival (RFS) and OS versus no irAEs, while grade 3-4 showed lesser benefit from RFS and no benefit from OS (Tarhini et al., 2021). Combined immunotherapy could induce more severe and sustained irAEs than monotherapy (Choi and Lee, 2020).

**FIGURE 2:**
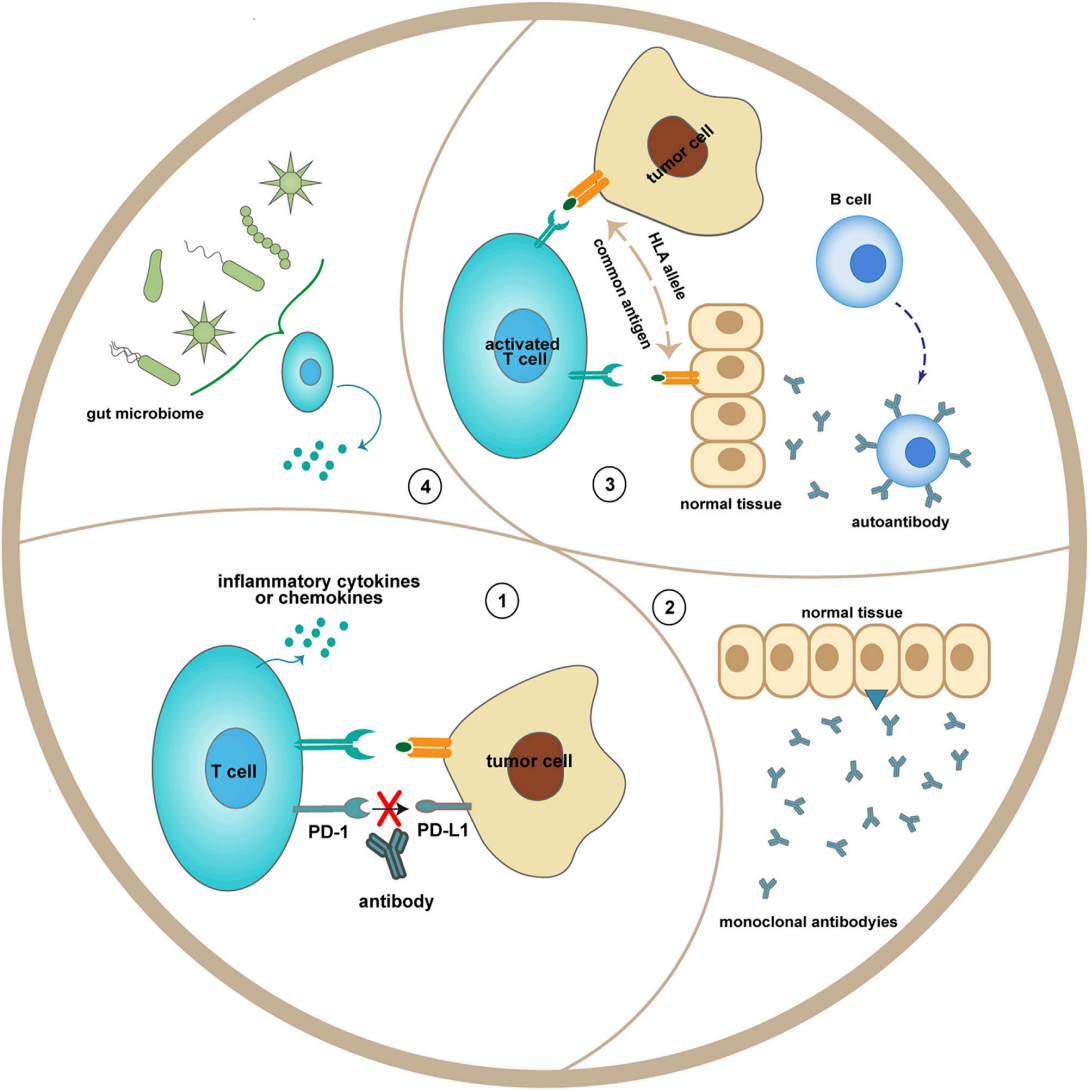
Potential mechanisms of immune-related adverse events. 1) Blocking the interaction between PD-1 on T cells and PD-L1 on tumor cells may enhance the release of inflammatory cytokines from T cells. 2) Monoclonal antibodies, like anti-CTLA-4, may recognize antigen presented by the normal tissue (hypothalamic and pituitary tissues). 3) Overresponse of naive lymphocytes could proliferate autoreactive T cells and B cells. 4) The gut microbiome, which may be altered after ICI treatment, may influence T-cell function.

T cells can undergo spontaneous differentiation into Tfh cells in CTLA-4-deficient mice, while not in CD28-deficient mice, they might be applied to explain lethal multiorgan autoimmune symptoms in *CTLA4−/−* mice (Walker, 2017). As precise mechanisms of irAEs have not been elucidated, some potential ones have been proposed: 1) Increased production of proinflammatory cytokines or chemokines can lead to immune-related damage in tissue which is anatomically prone. 2) Enhanced differentiation of lymphocytes containing T cells and B cells contributes to overpriming of T-cell-mediated immunity and overproduction of autoantibodies (Risbjerg et al., 2020; Ho et al., 2021). 3) Related to off-target effects of ICIs, hypophysitis induced by ipilimumab might be ascribed to targeting CTLA-4 expressed on pituitary tissues (Iwama et al., 2014). 4) The composition and percentage of the commensal microbiome may influence the curative effect for patients treated with ICI (Figure 2). The conclusion discovered from several kinds of research said that various irAEs were associated with the different superior microbiome, application of antibiotics was linked to poor prognosis, and fecal microbiota transplantation (FMT) could reduce immune colitis (Pierrard and Seront, 2019; Hommes et al., 2020; Andrews et al., 2021; Seton-Rogers, 2021). 5) Genetic susceptibility includes HLA haplotypes (Stamatouli et al., 2018).

For the treatment of irAEs, there have been some guidelines providing algorithms for most of the frequently occurring irAEs. 1) Before ICI initiation, patients’ condition should be evaluated, including family history, general physical condition, and baseline laboratory tests (Ramos-Casals et al., 2020). 2) For those suffering grade I or II irAEs in hardly lethal organs, they could continue/hold immunotherapy. Otherwise, they would better take immunosuppressive or immune-modulating drugs, including corticosteroids, as first-line medicine to control irAEs and relieve clinical symptoms (Esfahani et al., 2020). 3) For those who may bring irreversible or fatal consequences, it is necessary to withhold ICIs and apply steroids or other immunosuppressants immediately (Brahmer et al., 2021). 4) Individual basis should be taken into account when resuming discontinued ICIs owing to irAEs. There are also artificial solutions such as developing engineering antibodies that can induce responsive immune defense and limit systemic exposure of CTLA-4 blockade at the same time (Pai et al., 2019; Lacouture et al., 2021).

## Microbiome Related to ICI

With an estimated average of 3.8*10^13^ commensal bacterial resident in a 70 kg “reference man,” it is fluent in believing that gastrointestinal microbes play an important role in immunity (Sender et al., 2016). To date, there have been some oncogenic gut bacteria such as *Salmonella typhi*, *Helicobacter* spp., and *Helicobacter pylori* (Schwabe and Jobin, 2013; Gagnaire et al., 2017). On the contrary, some bacteria are thought to be beneficial for the proliferation of effector T cells and enhance antitumor efficacy (Pickard et al., 2017; Roy and Trinchieri, 2017). It is harder for mice supported in antibiotic exposed or germ-free conditions to benefit from CTLA-4 blockade versus those in specific pathogen-free environments (Vetizou et al., 2015). Thus, the linkage between microbiome and ICI needs to be elucidated (Table 2).

**TABLE 2:** The role of immune checkpoints in bacteria-related diseases.

Associated immune checkpoints	Associated bacteria/diseases	Related immune or other cells	Influence on efficacy	Mechanism	PMID
PD-L1	Oral administration of *Bifidobacterium* in melanoma	Antigen-specific CD8+ T cells	*Bifidobacterium*-treated mice showed a better antitumor effect compared to non-*Bifidobacterium*-treated mice	Oral administration of *Bifidobacterium* alone improved tumor control to the same degree as anti-PD-L1 therapy, and combination treatment nearly abolished tumor outgrowth. Augmented dendritic cell function leading to enhanced CD8+ T-cell priming and accumulation in the tumor microenvironment mediated the effect. Commensal *Bifidobacterium*-derived signals modulate the activation of DCs in the steady state, which in turn supports improved effector function of tumor-specific CD8+ T cells	26541606
PD-1	*Akkermansia muciniphila* in epithelial tumors	CCR9+CXCR3+CD4+ T lymphocytes	Enhancing the antitumor effect of PD-1 blockade	Oral gavages with *A. muciniphila* and *E. hirae* increased the efficacy of PD-1 blockade with respect to tumor growth and *A. muciniphila* and *E. hirae* induced dendritic cells to secrete IL-12, a Th1 cytokine involved in the immunogenicity of PD-1 blockade in eubiotic conditions. Oral supplementation with *A. muciniphila* after FMT with nonresponder feces restored the efficacy of PD-1 blockade in an interleukin-12-dependent manner by increasing the recruitment of CCR9+CXCR3+CD4+ T lymphocytes into mouse tumor beds	29097494
PD-1	*Faecalibacterium*	CD8+ T cell	Patients with high *Faecalibacterium* abundance had a significantly prolonged PFS versus those with a low abundance	Patients with a high abundance of Clostridiales, Ruminococcaceae, or *Faecalibacterium* in the gut had higher levels of effector CD4+ and CD8+ T cells in the systemic circulation with a preserved cytokine response to anti-PD-1 therapy, whereas patients with a higher abundance of Bacteroidales in the gut microbiome had higher levels of regulatory T cells (Treg) and myeloid-derived suppressor cells (MDSC) in the systemic circulation, with a blunted cytokine response	29097493
PD-L1	*Staphylococcus aureus* bacterial pneumonia in mouse model	CD4+ T cell; CD8+ T cell	Anti-PD-L1 therapy did not alter survival in this pneumonia model	Low dose- (LD-) or high dose- (HD-) SA: LD-SA and HD-SA produced lethality of 15 and 70% respectively by 168 h. At 24 h, LD-infected animals exhibited increased lung monocyte PD-L1 expression (*p* = 0.0002) but lower bacterial counts (*p* = 0.0002) compared to HD-animals. By 48 h, infection induced lung neutrophil or macrophage PD-L1 expression (*p* < 0.0001)	34009385
PD-1	*Mycobacterium tuberculosis* infection of rhesus macaques	Mtb-specific CD4 T cells	Animals treated with anti-PD-1 monoclonal antibody developed worse disease and higher granuloma bacterial loads compared	IFN-γ and TNF have both been previously implicated in increased growth of Mtb after PD-1 blockade. Inflammatory pathways (TNF; IFN-γ), normally important for host defense, are required for the exacerbation of Mtb infection after PD-1 blockade	33452107
PD-1	*Helicobacter pylori* in NSCLC	CD8+ T cell	*H. pylori* seropositivity associated with a decreased NSCLC patient OS and PFS on anti-PD-1 therapy	*H. pylori* seropositivity is associated with reduced effectiveness of anti-PD1 immunotherapy in patients with NSCLC. *H. pylori* infection affects not only DC function but also that of monocytes and/or macrophages. Indeed, in humans, it observed a decreased number of cells from the monocyte lineage and a substantially decreased expression of genes induced by type I interferon, IFN-γ, and IL-6 in the tumors of infected patients with NSCLC undergoing anti-PD1 treatment	34253574
PD-1	*Streptococcus pneumoniae* infection in mice	Pneumococcal capsule-specific B cells	PD-1 expression on B cells suppresses protective humoral immune responses to *Streptococcus pneumoniae*	B-cell-intrinsic PD-1 expression suppresses the protective humoral immune response to the capsule of *S*. *pneumoniae*. The selective suppression of TI-2 (T-cell-independent type 2) Ab responses by PD-1 interactions with B7-H1 and B7-DC points to a novel role for PD-1 in regulating Ag-specific B-cell responses to carbohydrate Ags	25624454
CTLA-4	*Faecalibacterium genus* and other *Firmicutes* in melanoma patients	Peripheral blood Tregs	Longer progression-free survival and overall survival and more frequent occurrence of ipilimumab-induced colitis	The Inducible T-cell COStimulator (ICOS) molecule is significantly upregulated on CD4^+^ T cells after ipilimumab (an immune checkpoint inhibitor (ICI) targeting CTLA-4) treatment in patients who belong to *Faecalibacterium*- driven cluster A	28368458
CTLA-4	Oral administration of *Bacteroides fragilis*, *Bacteroides thetaiotaomicron, Burkholderia cepacia*, or a combination of *B. fragilis* and *Burkholderia cepacia*	*B. fragilis*-specific T cells	Eliciting the antitumor immune response	The geodistribution of Bf (*B. fragilis*) in the mucosal layer of the intestine and its association with Burkholderiales recognized through the pyrin/caspase-1 inflammasome, synergizing with TLR2/TLR4 signaling pathways, may account for the immunomodulatory effects of anti-CTLA-4 Ab	26541610
CTLA-4	*Staphylococcus aureus* infection in mice	Low level of IL-6 production; high level of monocyte chemoattractant protein-1	It attenuates disease severity but may prolong the healing time required for *S. aureus* skin infections, having no impact on bacterial clearance in skin tissues	The pathogenic role of T-cell activation in certain *S. aureus* infections and the potential use of CTLA4 Ig to diminish tissue damage in those conditions	28264025
CTLA-4	*Mycobacterium tuberculosis* BCG infection	Enhances mycobacterial-infection-induced lymphocyte expansion and effector cell cytokine production in the draining lymph node but does not alter the number or function of lymphocytes at the primary site of infection	Enhancing immune response in the mediastinal lymph node with no improvement in clearance of mycobacteria in the lungs, liver, or spleen	CTLA- 4 blockade increased the antigen-specific expansion and differentiation of lymphocytes in the draining lymph node that is typically induced in response to a BCG lung infection	10417139
CTLA-4	*Helicobacter pylori* infection in mice	Regulation of balance between Th1 and Th2 response	Inhibition of the development of gastric inflammation, accompanied by an increasing ratio of *H. pylori*-specific IgG1/IgG2a in serum	The predominance of Th2 response by CTLA-4 blockade leads to an inhibition of the development of gastric inflammation. CTLA-4 signaling could contribute to the regulation of Th subsets and the development of gastric inflammation in *H. pylori* infection	14678261
CTLA-4	*Listeria monocytogenes* infection	*Listeria monocytogenes*-specific CD4+ and CD8+ T cells	Increasing numbers of CD4+ and CD8+ T cells and conferring stronger and rapid bacterial clearance	Blockade of CTLA-4 results in increased numbers of *L. monocytogenes*-specific CD4+ and CD8+ T cells after primary infection with attenuated *L. monocytogenes* and confers more rapid bacterial clearance after secondary challenge with virulent *L. monocytogenes*	19191906
CTLA-4	Mice infected with *Nippostrongylus brasiliensis*	T cells	Profound reduction in adult worm numbers and early termination of parasite egg production	The ability of CTLA-4 blockade to accelerate primary immune responses to a protective level during an acute infection indicates its potential as an immunotherapeutic tool for dealing with infectious agents	9221747
PD-1 and CTLA-4	Fungal sepsis in mice	Reverse sepsis-induced suppression of IFN-gamma and increased expression of MHC-II on APCs	Improving survival in bacterial sepsis	Blockade of cytotoxic T-lymphocyte antigen-4 (CTLA-4), a second negative costimulatory molecule that is upregulated in sepsis and acts like PD-1 to suppress T-cell function, also improved survival in fungal sepsis	23663657
LAG-3	*Mycobacterium tuberculosis*	CD4+ T cells and NK cells	Enhancing high bacterial burdens	Our data show that LAG-3 expressed primarily on CD4þ T cells, presumably by regulatory T cells but also by natural killer cells. The expression of LAG-3 coincides with high bacterial burdens and changes in the host type 1 helper T-cell response. LAG-3 marks a subpopulation of Tregs that are highly active and produce high levels of the cytokine IL-10, which are recruited to the lungs of primates with uncontrolled Mtb replication	25549835
LAG-3	*Mycobacterium tuberculosis*	CD4+ T cells	Modulating adaptive immunity	LAG-3 may modulate adaptive immunity to Mtb infection by interfering with the mitochondrial apoptosis pathway	28880895
LAG-3	*Staphylococcus aureus* and *Streptococcus pyogenes*	MAIT	Main coinhibitory molecule expressed by SEB-exposed MAIT cells	SEB-induced upregulation of LAG-3 on MAIT cells appears to rely on IL-12 and IL-18. SEB-induced MAIT cell anergy can be reversed by blocking LAG-3	28632753
LAG-3	*Plasmodium* parasites	T cell	A novel therapeutic strategy for this devastating infectious disease	Expression of the inhibitory receptors PD-1 and LAG-3 on CD4þ T cells and their reduced IL-2 production are common characteristic features of Plasmodium infection	26696540
LAG-3	Sepsis	CD4+ T cells; CD8+ T cells	Improving the survival and bacterial clearance in septic mice	LAG-3 was upregulated on CD4+ and CD8+ T cells, CD19+ B cells, natural killer cells, CD4+CD25+ regulatory T cells, and dendritic cells. Both LAG-3 knockout and anti-LAG-3 antibody had a positive effect on survival and on blood or peritoneal bacterial clearance in mice undergoing CLP. Cytokine levels and T-cell apoptosis decreased in anti-LAG-3 antibody-treated mice. Induced T-cell apoptosis decreased, whereas interferon γ secretion and proliferation were improved by anti-LAG-3 antibody *in vitro*. Interleukin 2 receptor was upregulated on T cells in both wild-type and LAG-3 knockout mice undergoing CLP	32347939
IDO1	*Mycobacterium tuberculosis*	Macrophage, CD141+ tolerogenic DCs, and myeloid-lineage cells	*M. tuberculosis* bacterial burden promotes dysregulated homing of CD4+ T cells in the T-cell zone of iBALT and poor restoration of CD4^+^ T cells in the lung interstitium	The macaque model of *M. tuberculosis* infection showed IDO-expressing cells in the macrophage-rich layer of granulomas, which likely serves to prevent optimal interactions between CD4+ T cells and *M. tuberculosis*-infected antigen-presenting cells (APCs). Moreover, increased expression of IDO1 correlated with *M. tuberculosis* bacterial burden, and IDO1 expression was also associated with poorly formed iBALT	32544085
IDO1	Autoimmune epididymitis	Plasmacytoid dendritic cells and regulatory T cells	Ido1 responds differently to autoimmune-mediated inflammation in the testis compared with the epididymis	IDO1 is known for its tolerogenic and immunosuppressive properties, exerted by modulating plasmacytoid dendritic cells and regulatory T cells. Ido1 responds differently to autoimmune-mediated inflammation in the testis compared with the epididymis	32383098
IDO1	*Coxiella burnetii*	Host cells	IDO1 production as a key cell-autonomous defense mechanism that limits infection by *C. burnetii*	IDO1 contributes to IFN-γ-mediated restriction of *C. burnetii*. IDO1 is an enzyme that catabolizes cellular tryptophan to kynurenine metabolites, thereby reducing tryptophan availability in cells. Cells deficient in IDO1 function were more permissive for *C. burnetii* replication when treated with IFN-γ, and supplementing IFN-γ-treated cells with tryptophan enhanced intracellular replication. Additionally, ectopic expression of IDO1 in host cells was sufficient to restrict replication of *C. burnetii* in the absence of IFN-γ signaling. Using differentiated THP1 macrophage-like cells, it was determined that IFN-γ activation resulted in IDO1 production and that supplementation of IFN-γ-activated THP1 cells with tryptophan enhanced *C. burnetii* replication	31461509
IDO1	*Mycobacterium tuberculosis*	Macrophages	IDO is associated with Mtb immune escape	Rv1737c is predominantly expressed by the Mtb in latent infection. In this study, we have characterized the Rv1737c functions in the recruitment and activation of macrophages, which play a cardinal role in innate and adaptive immunity. Rv1737c induced the tolerogenic phenotype of macrophages by upregulating the expression of indoleamine-2,3-dioxygenase 1 (IDO1)	31326120
IDO1	*Staphylococcus aureus* and *Toxoplasma gondii*	Human retinal pigment epithelial (hRPE) cells	Inhibiting the growth of *T. gondii* and *S. aureus*	We found that an IFN-γ stimulation of hPRE cells induced the expression of IDO1, which inhibited the growth of T. gondii and *S. aureus*. Costimulation with IFN-γ, interleukin-1 beta, and tumor necrosis factor alpha induced a strong expression of iNOS. The iNOS-derived nitric oxide production was dependent on cell-culture conditions; however, it could not cause antimicrobial effects. iNOS did not act synergistically with IDO1. Instead, iNOS activity inhibited IDO1-mediated tryptophan degradation and bacteriostasis	31267172
IDO1	*Chlamydia trachomatis*	Human endometrial carcinoma cell line; peripheral blood mononuclear cells	IDO1 catalyzes the degradation of tryptophan, which can eliminate *C. trachomatis* infection *in vitro*	In PBMCs infected with *C. trachomatis* there was a significant upregulation in IDO1 levels, which was independent of IFN-γ. In fact, *C. trachomatis* infection in PBMCs failed to induce IFN-γ levels in comparison to the uninfected culture	30832593
IDO1	Uropathogenic *Escherichia coli*	epithelial cell	IDO1 activity regulates PMN chemotaxis in response to epithelial bacterial infection	The idea of an expanded role for IDO in innate cellular responses through the AHR-mediated effects of kynurenine metabolites on neutrophil function, in addition to the previously identified roles in adaptive immune regulation	26857571
IDO1	*Candida albicans*	Treg	Contributing to establish immune tolerance and allow fungi colonization	Subsequent inflammatory Th1-type immunity was modulated by induced Treg cells, which required the TRIF pathway as well, and acted through activation of IDO in dendritic cells and Th17 cell antagonism (17947673)	17947673; 15728500
IDO1	*Aspergillus* species	Treg	Controlling fungal burdens of Aspergillus species by activating distinct populations of Treg cells	*S. cerevisiae* has only one IDO gene (BNA2) and, to date, it has only been associated with one function, NAD + synthesis	21170645
IDO1	A primary fungal in pulmonary paracoccidioidomycosis	T cell	Controlling fungal loads and immunity, the impairment of IDO1 activity could play a major role in the pathogenesis of severe forms of human pulmonary	IDO inhibition was shown to induce increased fungal loads in resistant and susceptible mice concomitantly with increased induction of NO synthesis	25411790
VISTA	Experimental autoimmune encephalomyelitis	CD4+ effector T cells; CD8+ effector T cells	Treatment with VISTA-blocking mAb led to more severe disease in the EAE mode	VISTA overexpression on tumor cells interferes with protective antitumor immunity *in vivo* in mice. These findings show that VISTA, a novel immunoregulatory molecule, has functional activities that are nonredundant with other Ig superfamily members and may play a role in the development of autoimmunity and immune surveillance in cancer	21383057
TIM-3	*Mycobacterium tuberculosis*	Macrophage	TIM-3-immunoglobulin fusion protein reduced the *M. tuberculosis* burden in M*. tuberculosis*-infected mice. TIM-3/Gal-9 pathway in triggering antibacterial activity in *M. tuberculosis*-infected human macrophages	The TH1 cell surface molecule TIM-3 has evolved to inhibit the growth of intracellular pathogens via its ligand Gal9, which in turn inhibits expansion of effector TH1 cells to prevent further tissue inflammation	20937702; 23180810

Clinical studies have reported that bacterial species can be differentially abundant in responders versus nonresponders (Katayama et al., 2019). Through feeding with *B. fragilis*, immunization with *B. fragilis* polysaccharides, or adoptive *B. fragilis*-specific T cells transfer, mice that failed in CTLA-4 blockade could regain their immunity. Transplantation of microbiota from melanoma patients to mice proved that *B. fragilis* favored the CTLA-4 blockade (Vetizou et al., 2015). In metastatic melanoma, Chaput et al. reported that patients with enriched *Faecalibacterium* and other *Firmicutes* as baseline microbiota presented a better prognosis than those with *Bacteroides*. However, the *Bacteroidetes* bring little colitis than *Faecalibacterium* (Chaput et al., 2017). In linkage with this, Gopalakrishnan et al. discovered that *Faecalibacterium* was enriched in responders, while *Bacteroides thetaiotaomicron* was enriched in nonresponders in melanoma patients (Gopalakrishnan et al., 2018). Using 16S ribosomal RNA gene sequencing, Matson et al. found out that *Bifidobacterium longum*, *Collinsella aerofaciens*, and *Enterococcus faecium* were more abundant in anti-PD-1 responders with metastatic melanoma (Matson et al., 2018).

The potential mechanisms through which the immune response is regulated by the microbiome may be as follows (Mazmanian et al., 2005; Helmink et al., 2019; Hayase and Jenq, 2021): 1) Through linkage between PAMPs and pattern recognized receptors (PRRs, such as Toll-like receptors), the adaptive immune response can be activated by APCs. 2) Cancer cells can bear cross-reactive neoantigens with microbiota, thus inducing an immune response. 3) Cytokines secreted by APCs or lymphocytes can be altered with specific metabolites or bacterial byproducts. 4) Metabolites entering the bloodstream could elicit a systemic response.

It also has been reported that irAEs induced by CTLA-4 occur most commonly and frequently at sites of the GI tract rich in bacteria. Disrupting the gut microbiota via antibiotics could potentially impair antitumor immune responses as well as response to immune checkpoint blockade (Helmink et al., 2019). Reconstruction of GI microbiome using FMT from healthy or responding donors shows a promising therapeutic effect with ICI-associated colitis relief and proportion of Tregs increase (Wang et al., 2018).

Still, the limitations of FMT should be taken into consideration. The connection between favorable microbiota and certain immune checkpoint blockade needs to be cleared. There could be adverse events induced by FMT, as we talked about above in IrAEs, either.

## Immune Checkpoint Molecules in Virus-Infected Diseases

In chronic viral infection and cancer, due to long-term and low magnitude exposure to antigen, that T cell progressively loses its effector function with elevated coinhibitory receptor constitutive expression in order to diminish tissue damage is called “T-cell exhaustion.” Many pathogens and cancers promote inhibitory interactions to escape immune surveillance (Table 3). Thus, reversing the T-cell state is regarded as an effective solution in infectious diseases.

**TABLE 3:** The role of immune checkpoints in virus infection diseases.

Associated immune checkpoints	Associated virus	Associated diseases	Related immune cells or other cells	Influence on efficacy	Mechanism	PMID
PD-1	HBV	Acute or chronic HBV infection disease	HBsAg-specific B cell unable to mature into Ab-secreting cells and displayed increased expression of CD21lo and PD-1	Anti-PD-1 antibodies could partially restore HBsAg-specific B-cell maturation	HBV infection has a marked impact on global and HBV-specific humoral immunity, yet HBsAg-specific B cells are amenable to a partial rescue by B-cell maturing cytokines and PD-1 blockade	30084841
PD-1	HCV	Chronic HCV-infected chimpanzees	Restoring intrahepatic CD4+ and CD8+ T-cell immunity	Significant reduction in HCV viremia in responder animal	Successful PD-1 blockade likely requires a critical threshold of preexisting virus-specific T cells in liver and warrants consideration of therapeutic vaccination strategies in combination with PD-1 blockade to broaden narrow responses	23980172
PD-1	HIV	HIV-infected patients	Increasing CD8+ T cells in patients with chronic HIV infection	Cof blocking CD39/adenosine and PD-1 signaling showed a synergic effect in restoring CD8+ T-cell function (secrete functional cytokines and kill autologous reservoir cells) *in vitro*	Combined blockade of CD39/adenosine and PD-1 signaling *in vitro* may exert a synergistic effect in restoring CD8+ T-cell function in HIV-1-infected patients	34177939
PD-1	CMV	Chronic CMV infection after renal transplantation	Higher positive rate of PD-1 in CMV-specific CD4+ T cell from viremic transplant recipients, loss of IL-2 production	Blockade of PD-1/PD-L1 could reverse functional anergy of CMV-specific CD4+ T cell and increase 10-fold proliferation in CMV-specific CD4+ T cell	Expression of PD-1 defines a reversible defect of CMV-specific CD4 T cells that are associated with viremia, and blocking PD-1 signaling may provide a potential target for enhancing the function of exhausted T cells in chronic CMV infection	18510628
PD-1	HPV	HPV associated squamous cell carcinoma of the head and neck	Cancer cells	Pembrolizumab was tolerated with 17% grade 3-4 irAEs; the overall response was 25% in HPV-positive patients	Greater antitumor activity was recorded in patients with squamous cell carcinoma tumors of the head and neck that expressed higher levels of PD-L1 and interferon-γ-related genes. Thus, pembrolizumab might represent a new treatment approach for patients with squamous cell carcinoma of the head and neck	27247226
CTLA-4	LCMV	Mice chronically infected with LCMV	Virus-specific CD8+ T cell	Blockade of the CTLA-4 had no effect on either T-cell function or viral control	Inhibition mediated by PD-1 requires close proximity of PD-1 to the site of TCR engagement and does not signal in the absence of a TCR signal. Following crosslinking by PD-1 ligand, the immunoreceptor tyrosine-based switch motif (ITSM) in the cytoplasmic domain of PD-1 is phosphorylated and recruits the phosphatases SHP-1 and SHP-2. These phosphatases act on proximal signaling kinases of the TCR pathway, reducing the TCR signal and leading to diminished T-cell activation and cytokine production. Therefore, under conditions of persistent antigen, T cells may modulate their responsiveness by upregulating inhibitory receptors such as PD-1 that attenuate TCR signaling	16382236
CTLA-4	HBV	Chronic HBV infection	CTLA-4 is upregulated on HBV-specific CD8+ T cells with the highest level of Bim protein	Blocking CTLA-4 can increase the expansion of IFN-gamma producing HBV-specific CD8+ T cells	CTLA-4 is expressed by HBV-specific CD8+ T cells with high levels of Bim and helps to drive this proapoptotic phenotype	21360567
CTLA-4	HCV	Patients with hepatocellular carcinoma and chronic HCV infection	Cancer cells	Anti-CTLA-4 showed a good safety profile; no patients needed steroids due to severe irAEs; disease control rate was 76.4%	HCV-specific CD8+ T cells that are exhausted express various inhibitory receptors, including CTLA-4 that acts synergistically with the programmed cell death-1 receptor (PD-1) to enforce their exhaustion state. Moreover, CTLA-4 is preferentially upregulated in PD-1+ T cells from the liver of chronically HCV-infected patients. It seems possible that the revival of antiviral T-cell immunity in patients with long-lasting chronic HCV infection following tremelimumab therapy may result from increased CD4+ T cell help and recovery of CD8+ T-cell exhaustion	23466307
CTLA-4	HIV	HIV-infected patients	No pattern was noted regarding the change from baseline in CD4 or CD8 T cells	No serious adverse events or dose-limiting toxicities and ipilimumab were associated with variations in HIV RNA	Ipilimumab treatment of an HIV-infected patient on antiretroviral therapy increased CD4+ T cells, predominantly total memory and effector-memory cells, postinfusion along with transient increases in CD8+ T cells without change in cell activation. Furthermore, ipilimumab increased cell-associated unspliced HIV RNA and a subsequent decline in plasma HIV RNA	29879143
CTLA-4	HIV	HIV-infected patients	CTLA-4 was upregulated in HIV-specific CD4+ T cells but not CD8+ T cells	CTLA-4 expression correlated positively with disease progression and negatively with the capacity of CD4+ T cells to produce interleukin 2 in response to viral antigen. *In vitro* blockade of CTLA-4 augmented HIV-specific CD4+ T-cell function	CTLA-4 ligation can suppress effector T-cell functions both directly through CTLA-4 expressed on effector cells and indirectly through CTLA-4 expressed on CD4+CD25+ Treg cells. A CTLA-4-mediated effect of Treg cells can probably occur *in vivo* both by direct T-cell-T-cell contact and indirectly by induction of indoleamine-2,3-dioxygenase in dendritic cells	17906628
PD-1/CTLA-4	HAV	HAV-associated hepatitis	Isolated PBMC, PD-1, and CTLA-4 on T cells were measured by flow cytometry	Significantly higher expression of PD-1 and CTLA-4 on T cells consistent with a viral-protective effect of PD-1 and CTLA-4, thereby preventing the destruction of virus-infected hepatocytes in AHA	The changing expression of PD-1 and CTLA-4 during the symptomatic and recovery phases of AHA points to the protective effects of these inhibitory molecules, perhaps by suppressing the activity of cytotoxic T cells, thereby preventing the induced fulminant destruction of HAV-infected hepatocytes	26347518
PD-1/CTLA-4	EBV	Intraperitoneally inject EBV-infected human cord blood into NSG mice	Increasing EBV-specific T-cell response and enhancing tumor infiltration by CD4+ and CD8+ T cells	Combination of PD-1/CTLA-4 blockade reduced the size of lymphoma, decreased the number of both latently and lytically EBV-infected B cells	PD-1/CTLA-4 blockade markedly increases EBV-specific T-cell responses and is associated with enhanced tumor infiltration by CD4+ and CD8+ T cells	27186886
PD-1/CTLA-4	SIV	SIV-infected long-term antiretroviral therapy-treated rhesus macaques	Decreasing total and intact SIV-DNA in CD4+ T cells and B-cell follicles	Inducing robust latency reversal and reducing total levels of integrated virus. No enhanced SIV-specific CD8+ T-cell responses or viral control	Dual CTLA-4/PD-1 blockade produced a significant reduction in cell-associated SIV-DNA within LN CD4+ TEM, the CD4+ T-cell subpopulation most activated from combined treatment. Importantly, *in situ* hybridization assays demonstrated a significant reduction in the number of vRNA+ and vDNA + cells following dual CTLA-4/PD-1 blockade in the LN, including in the BCF	32284611
LAG-3	HIV	AIDS	T cell	High viral load, faster disease progression, and rapid return of viremia following treatment interruption	Although mechanisms and functions of LAG-3 remain controversial, LAG-3 clearly inhibits immune responses. If LAG-3 blockade improves immune function during HIV infection, it could help deplete the HIV reservoir by reversing latency and restoring immunity of exhausted cells	30653605
LAG-3	HBV	Hepatocellular carcinoma	CD8 (+) T cells	Acting as a suppressor of HBV-specific	Since LAG-3 is an inhibitory molecule that plays a downregulatory role on T-cell responses, we found the correlation between LAG-3 expression and HBV-specific CD8+ T cells dysfunction	23261718
LAG-3	HPV	OPSCC	CD8 (+) T cells	HPV-related OPSCC might be more susceptible to single or combined anti-LAG-3 antibody therapy than HPV-negative OPSCC patients	Possible reasons for this may be the interrelationship of multiple components in the tumor immune microenvironment, as it has been reported that the coexpression of LAG-3 with other inhibitory molecules such as TIM-3 or PD-1 induces the exhaustion of immune cells, resulting in downregulated cytokine expression	33396515
LAG-3	HCV	Follicular lymphoma	CD8 T cells	Inhibiting cell proliferation, cytotoxicity function, and cytokine production	LAG-3 expression could be substantially upregulated on CD4+ or CD8+ T cells by IL-12, a cytokine that has been shown to induce T-cell exhaustion and be increased in the serum of lymphoma patients. Furthermore, we found that blockade of both PD-1 and LAG-3 signaling enhanced the function of intratumoral CD8+ T cells resulting in increased IFN-γ and IL-2 production	28977875
LAG-3	LCMV	Chronic viral infections	CD8 T cells	LAG-3 is continuously upregulated on LCMV-specific exhausted CD8 T cells; it alone does not significantly contribute to T-cell exhaustion	LAG-3 is upregulated on LCMV-specific exhausted CD8 T cells; it does not significantly contribute to T-cell exhaustion alone. To effectively interfere with T-cell exhaustion, it is very likely that several inhibitory receptors will have to be targeted simultaneously	19880580
IDO1	HIV	HIV-1 infection	CD4+ T cells	IDO may represent a critical initiating event that results in inversion of the T(H)17/T (reg) balance and in the consequent maintenance of a chronic inflammatory state in progressive HIV disease	IDO1-dependent tryptophan catabolism may be an important link between immune activation and the gradual decline of immune function seen in progressive HIV infection	20484731
IDO1	HPV16	Head and neck squamous cell carcinomas	HPV16-specific CD8+ T cells	The HPV16 CTL epitopes identified in this study, in combination with blockade of HPV + HNSCC-specific PD-1/IDO-1 checkpoints, may be useful for targeted immunotherapy	Our findings implicate mechanisms of T-cell escape in HPV + HNSCC, wherein high tumoral HPV-antigen load results in high expression of immune dysfunction genes on tumor cells (e.g., IDO-1) and dysfunction of HPV-specific CTLs (e.g., E7; E2-CTLs). HPV + HNSCCs expressing IDO-1 might similarly be driven by HPV-specific-CTL infiltration in response to high tumoral HPV-antigen load	30154146
IDO1	HPV	Chronic infection	Invariant natural killer T; T cell	Induction of IDO1 in HPV-infected skin contributes to evasion of host immunity	Inhibiting IDO activity using 1-methyl-DL-tryptophan (1-D/L-MT) promotes K14E7 skin graft rejection. Increased IDO1 expression and activity in K14E7 skin require IFN-g and invariant natural killer T (iNKT) cells, both of which have been shown to negatively regulate T-cell effector function and suppress K14E7 graft rejection. Furthermore, DCs from K14E7 skin express higher levels of IFN-g receptor (IFN-gR) than DCs from control skin	23652797
VISTA	HIV	AIDS	CD4+ and CD8+ T cells	Gal-9 and VISTA expression was associated with impaired T-cell effector functions	A dramatic reduction in the production of cytokines by T cells expressing PD-1, CD160, CD39, TIM-3, and VISTA. In contrast to other coinhibitory molecules, the pattern of cytokine production was not different between 2B4+ and 2B42 CD4+ T cells, and interestingly 2B4+ CD8+ T cells exhibited higher cytokine production capabilities compared with 2B42 CD8+ T cells	32205423
TIM-3	HIV	AIDS	T cells	Blocking the TIM-3 signaling pathway restored proliferation and enhanced cytokine production in HIV-1-specific T cells	In progressive HIV-1 infection, TIM-3 expression was upregulated on HIV-1-specific CD8 + T cells. TIM-3-expressing T cells failed to produce cytokine or proliferate in response to antigen and exhibited impaired Stat5, Erk1/2, and p38 signaling. Blocking the TIM-3 signaling pathway restored proliferation and enhanced cytokine production in HIV-1-specific T cells	19001139
TIM-3	HCV	HCV infection	HCV-specific CTLs	Blockade of either PD-1 or TIM-3 enhanced *in vitro* proliferation of HCV-specific CTLs to a similar extent, whereas cytotoxicity against a hepatocyte cell line that expressed cognate HCV epitopes was increased exclusively by TIM-3 blockade	Early accumulation of PD-1+TIM-3+ T cells is associated with functional impairment and consequently with the development of persistent HCV. The present study provides a basis for improving current therapies by simultaneous blockade of multiple inhibitory pathways that could result in additive efficacy without excessive toxicity	21084749
TIM-3	LCMV	Chronic LCMV infection	CD8 T cell	Targeting both PD-1 and TIM-3 is an effective immune strategy for treating chronic viral infections	Whereas TIM-3 was only transiently expressed by CD8 T cells after acute infection, virus-specific CD8 T cells retained high TIM-3 expression throughout chronic infection. The majority (approximately 65–80%) of lymphocytic choriomeningitis virus-specific CD8 T cells in lymphoid and nonlymphoid organs coexpressed TIM-3 and PD-1. This coexpression of TIM-3 and PD-1 was associated with more severe CD8 T-cell exhaustion in terms of proliferation and secretion of effector cytokines such as IFN-γ, TNF-α, and IL-2. Interestingly, CD8 T cells expressing both inhibitory receptors also produced the suppressive cytokine IL-10. Most importantly, combined blockade of TIM-3 and PD-1 pathways *in vivo* synergistically improved CD8 T-cell responses and viral control in chronically infected mice	20679213
TIM-3	Friend virus	Acute Friend virus-induced disease	CD8 T cell	Combined blockade of PD-1 and TIM-3 during the priming/differentiation phase rescued FV-specific CD8 (+) T cells from becoming terminally exhausted, resulting in improved CD8 (+) T-cell functionality and virus control	TIM-3 and CTLA-4 were recently found to be overexpressed on HIV- and hepatitis C virus-specific CD4+ and CD8+ T cells and to act to suppress effector functions of activated T cells. Upregulation of LAG-3 was also shown to correlate with the impaired effector functions and exhaustion of CD8+ T cells	20351188
TIM-3	HBV	Chronic HBV infection	CD4+ and CD8+ T cells	Overexpression of TIM-3 is involved in disease progression of CHB and that TIM-3 may participate in skewing of Th1/Tc1 response, which contributes to the persistency of HBV infection	The expression of TIM-3 is upregulated on circulating CD4+ and CD8+ T cells in CHB patients. TIM-3 was highly expressed on T cells from AHB patients as well; however, its expression decreased dynamically in the convalescence phase. TIM-3 expression positively correlated with disease severity and negatively correlated with Th1/Tc1 response in CHB patients	21392402

In mice with chronic LCMV infection, blockade of PD-1 restored CD8+ T cell function, suggesting that T-cell exhaustion is reversible. In patients with chronic hepatitis B, CTLA-4 blockade can reinvigorate hepatitis B virus- (HBV-) specific CD8+ T cells in both intrahepatic and peripheral compartments (Cao et al., 2018). With the coinhibition of PD-1 and CTLA-4, the effector function of HCV-specific CD8+ T cells can be restored in chronic hepatitis C patients (Cho et al., 2017). Meanwhile, inhibition of PD-1 can induce the production of cytokines (e.g., IFN-γ) in HIV/HBV-specific CD8+ T cells to enhance immune response (Jubel et al., 2020). Coexpressing with PD-1, LAG-3, TIM-3, and TIGIT blockade can also reverse dysfunctional T-cell responses and reduce cytokines production. It is widely known that TIM-3 is highly upregulated on virus and tumor Ag-specific CD8+ T cells, and antagonizing TIM-3 helps restore the function of CD8+ T cells (Clayton et al., 2014). Expression of LAG-3 has been reported to be associated with a reduction in invariant NKTs IFN-γ production during chronic HIV infection (Juno et al., 2015).

## Discussion and Future Perspectives

**FIGURE 3:**
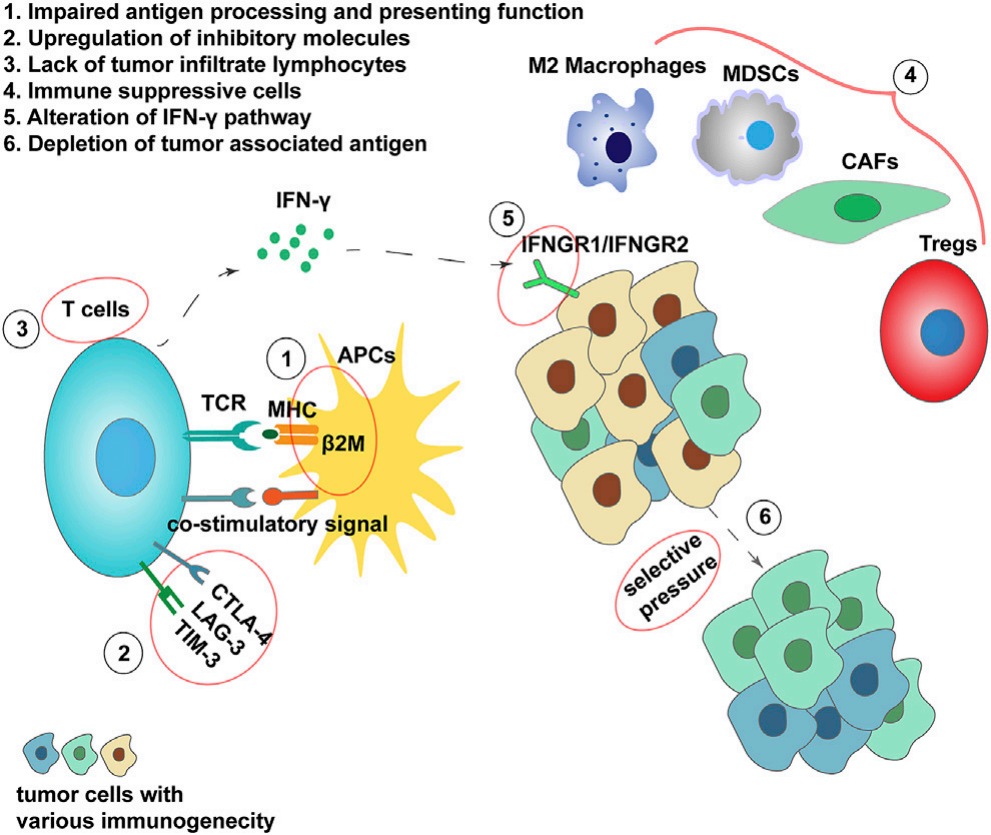
Mechanisms of resistance from ICI treatment. 1) β2M mutations lead to loss of HLA and antigen-presenting function. 2) Additional inhibitory signals expression. 3) Little tumor-infiltrating lymphocytes present in the tumor microenvironment resulting in nonresponse. 4) Immune suppressive cells in TME. 5) Loss of IFN-γ sensitivity. 6) Formation of low immunogenicity clone under selective pressure.

Immune checkpoints are some vital regulators of the immune system. Now in most referred contexts, immune checkpoints are equivalent to inhibitor regulators of the immune system. Despite the immune checkpoint molecules that we have discussed above, there are still other immune checkpoint molecules, such as BTLA, KIR, A2AR, B7-H4, NOX2, HO-1, and SIGLEC7. Besides, the stimulatory immune checkpoints are also promising targets for immune therapy, such as CD40, CD122, CD137, OX40, and GITR. Relying on neoantigen expressed on tumor cells, T cells can target and exclude potential threats. So as to escape from host immunity, tumor cells requisition inhibitory molecules to bind and silence immune cells. The availability of immune checkpoint blockade as one of the effective supplemental methods for tumor treatment has been verified. However, some tumors show low immunogenicity and cannot respond effectively to immune checkpoint blockade. For initially responding tumors, selection of low immunogenic clones and inducement of tolerance due to tumor heterogeneity will develop frequent relapses and even hyperprogression in nonresponders, of which the range was between 4 and 29% (Denis et al., 2020). Such phenomenon is known as resistance (Sharma et al., 2017). The mechanisms of resistance can be divided into intrinsic and extrinsic (Figure 3). The intrinsic mechanisms are composed of lack of tumor antigen presentation, alteration of several inhibitory signaling pathways, and upregulation of other immune checkpoints. The extrinsic mechanisms are predominantly referred to as various elements in the TME (Baxter et al., 2021). To reverse the resistance and ameliorate patients’ symptoms, researchers came up with the idea to turn the “cold” immune response to “hot.” The strategies applied under such fundamental idea consist of turning down the volume of inhibitory immune signals, triggering T-cell priming, increasing the costimulatory signals, and modulation of the TME (Attili et al., 2021; Weiss and Sznol, 2021).

Meanwhile, the sailing of drug development is never smooth. Hundreds of clinical trials to develop new agents targeted at immune checkpoints have been terminated due to low responsiveness and fatal irAEs. IrAEs induced by ICI are an impassable mountain lying in front of us, with death as the most severe consequence. The clinical trial testing sym022 (anti-LAG-3 mAb) in humans with metastatic cancer, solid tumors, or lymphoma exhibits an unwanted outcome with high progression and irAEs rate (NCT03489369). In addition, the mechanisms under ICI still need to be shed light on.

In conclusion, despite the shortcomings of immune checkpoint blockade in clinical application, it is a promising strategy for cancer therapy, with a considerable proportion of applicants achieving an objective response. Further studies are needed to be explored to elucidate precise mechanisms, achieve potential will, and ameliorate adverse events to benefit more patients with tumors and other diseases.
